# Renal and mediastinal perivascular epithelioid cell tumors (PEComas) in a young child with tuberous sclerosis; a rare case report

**DOI:** 10.1259/bjrcr.20220105

**Published:** 2022-12-21

**Authors:** Kamal H. Attia, Ahmad A. Al Boukai, Muammar H. Mohammed, Maha Arafah

**Affiliations:** 1 Department of Radiology and Medical Imaging, King Saud university medical city, Riyadh, Saudi Arabia; 2 Department of Pathology, King Saud University, Riyadh, Saudi Arabia

## Abstract

We describe a unique case of perivascular epithelioid cell tumors occurring as mediastinal and left renal soft tissue masses discovered incidentally in a 5-year-old tuberous sclerosis patient upon presentation to the emergency department for upper respiratory illness. The radiographic features were non-specific. However, the similar CT characteristics of both lesions and background history raised the suspicion of a synchronous mesenchymal tumor, and histopathology confirmed the diagnosis. The rarity of these tumors in the pediatric population and lack of specific diagnostic criteria impose reporting the case and emphasize the need for further research on imaging features of such tumors.

## Case presentation

A 5-year-old boy, a known case of tuberous sclerosis, presented to our emergency department (ED) complaining of a runny nose, cough, and fever for 1 week. Initial physical examination revealed congested tonsils and decreased air entry in the right lung. No chest compressive symptoms or remarkable abdominal complaints.

## Investigations

**Figure 1.**
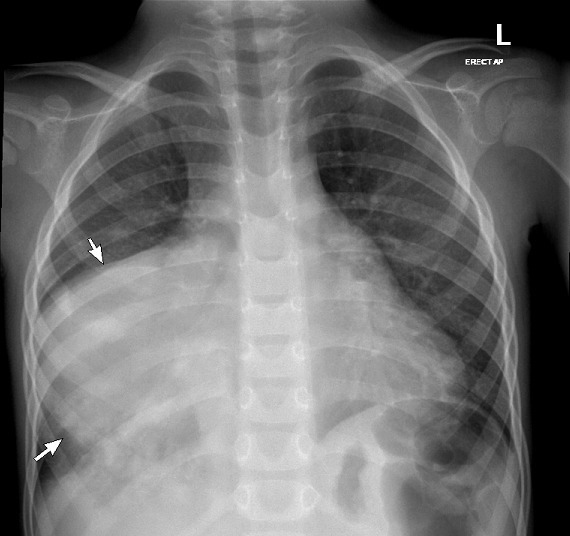
Chest X-ray showing a large mass in the right lower hemithorax with broad mediastinal attachment (arrows). Thickening of the right para-tracheal stripe that could be related to paratracheal lymphadenopathy.

**Figure 2.**
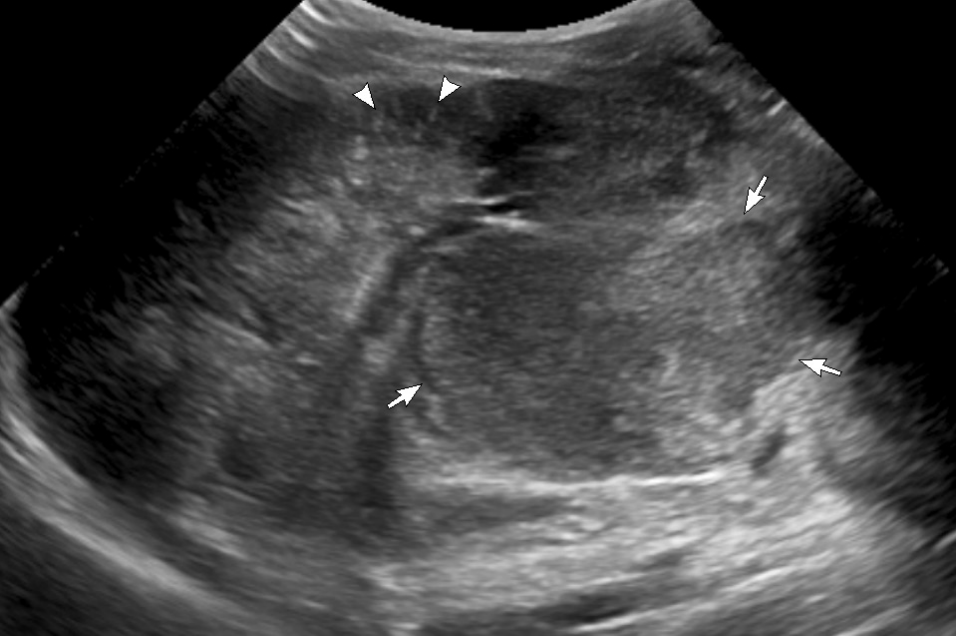
A selected ultrasound image of the left kidney showing the sizeable exophytic mass arising from the lower pole (arrows) and multiple tiny cortical AMLs (arrowheads). AML, angiomyolipoma.

Chest X-ray showed a large mass in the right lower chest with broad base mediastinal attachment and multiple small nodular and tubular infiltrate in the right lower lobe(Figure 1). Ultrasound of the abdomen and pelvis revealed a sizeable exophytic mass arising from the lower pole of the left kidney measuring approximately 6 × 7 cm (Figure 2). Enumerable small hyperechoic lesions in both renal cortices and several hyperechoic liver lesions represent renal angiomyolipoma (AML) and hepatic lipomas/AMLs.

**Figure 3.**
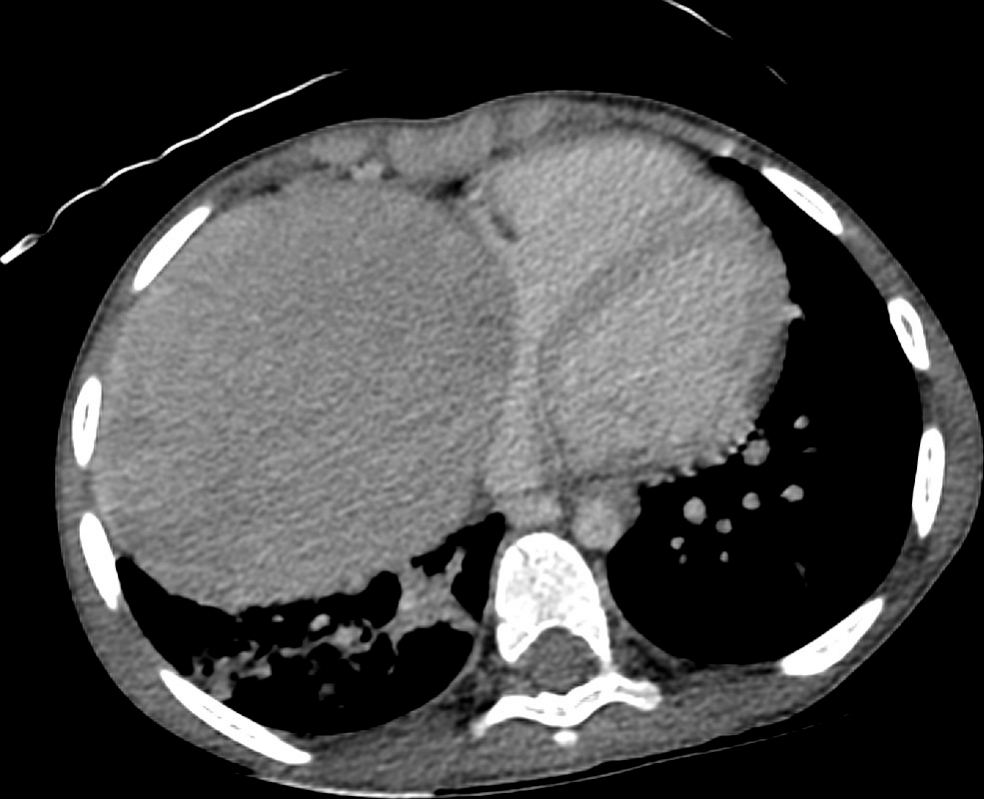
CECT of the chest showing the large anterior mediastinal mass, inseparable from the right-side cardiac border and atelectatic changes in the right lower lung lobe. CECT, contrast-enhanced CT.

**Figure 4.**
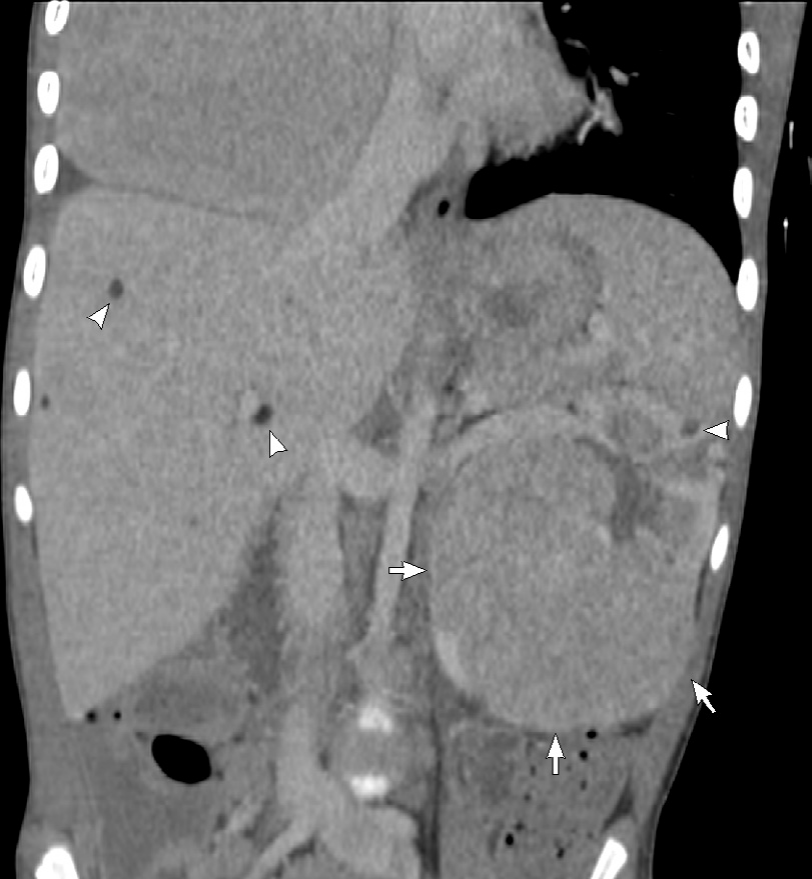
CECT of the abdomen in coronal reformat showing the exophytic left renal mass (arrows) causing mild mass effect upon left renal vein and partially visualized anterior mediastinal mass. Note the attenuation similarity of both masses. There are multiple small left renal AMLs and hepatic lipomas/AMLs(arrowheads). AML, angiomyolipoma; CECT, contrast-enhanced CT.

A contrast-enhanced CT scan of the chest, abdomen, and pelvis was performed. The chest mass had a well-demarcated outline, originating from the anterior mediastinum and inseparable from the right cardiac chambers (Figure 3). It measures 11 × 9 x 8 cm. The exophytic left renal mass in the abdomen appeared to have a lobulated contour measuring approximately 9.5 × 7 x 3.5 cm (Figure 4) with no extension beyond the renal capsule. Both masses had relatively high attenuation (≈60 HU) in the pre-contrast phase and moderate inhomogeneous enhancement (≈97 HU) in the arterial phase, and the attenuation decreased by 20 HU in the delayed phase. Both masses had no visible fat, calcifications, or cystic degeneration. There were multiple enlarged mediastinal lymph nodes but no abdominal or pelvic lymphadenopathy. Contrast-enhanced brain MRI showed numerous cortical and subcortical tubers, multiple subependymal hamartomas, and an enhancing lesion at the left foramen of Monro measuring 1.2 × 1 cm representing subependymal giant cell astrocytoma (SEGA).

An ultrasound-guided true-cut biopsy from both masses was performed. Routine H&E staining and extensive immunohistochemistry analysis, including HMB45, Melan-A, desmin, SMA, S-100, LMWCK, CK7, and RCC markers confirmed the diagnosis of epithelioid angiomyolipoma.

## Differential diagnosis

For the left renal mass, renal cell carcinoma should be included in the differential diagnosis due to increase risk in TSC patients’, although, it’s rare to occur at that age. Lymphoma is also important differential diagnosis combining both lesions.

## Diagnosis

The diagnosis of benign perivascular epithelioid cell tumors was established based on extensive immunohistopathology analysis.

## Treatment and outcome

**Figure 5.**
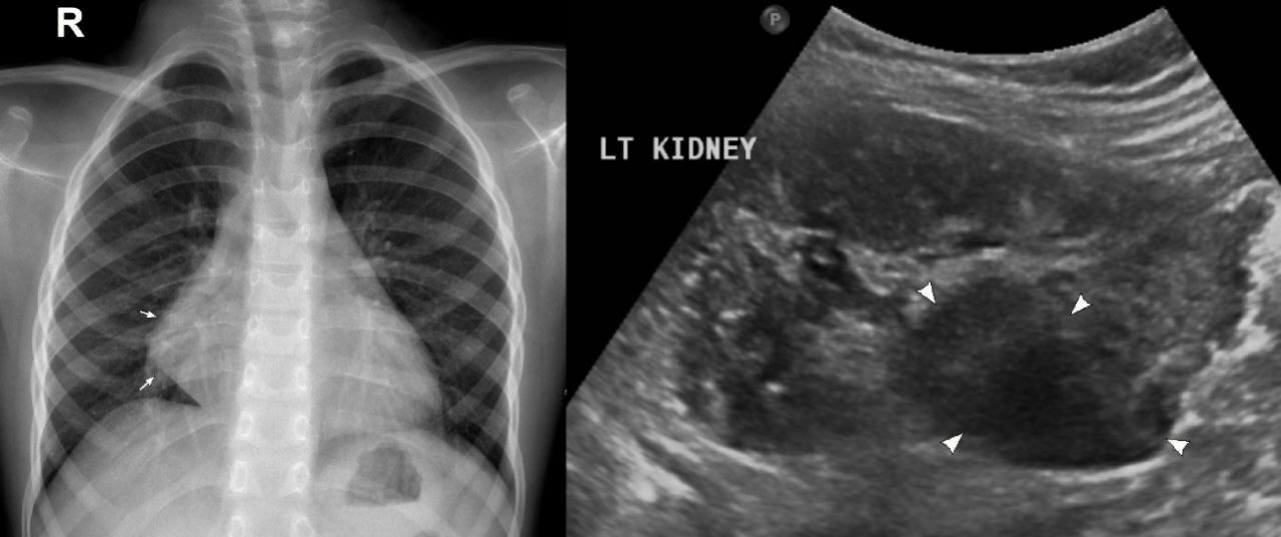
Follow-up chest X-ray and ultrasound of left kidney after approximately 2 years of medical treatment showing significant size reduction of the mediastinal (arrows) and renal (arrowheads) masses.

The multidisciplinary team (MDT) discussed the case thoroughly, and the group agreed on conservative management because the lesions were incidentally discovered despite the large size, histopathological similarities, and no malignant features. An mTOR inhibitor (Sirolimus 3 mg daily) was commenced with a significant progressive decrease in the size of both lesions throughout follow-ups. (Figure 5a and b)

## Discussion

Perivascular epithelioid cell tumors (PEComas) are defined as “mesenchymal tumors composed of histologically and immunohistochemically distinctive perivascular epithelioid cells (PECs)," according to WHO definition in 2002.^1^ They were first presented by Bonetti et al in 1992, and 4 years later, the term (PEComa) was proposed by Zamboni.^1^ PEComas have been described in various organs such as kidney, liver, pancreas, genital organs, bladder, and other visceral and soft-tissue sites.^2,3^

PEComas of the kidney have a broad spectrum of histologic subtypes, including classic AML, epithelioid AML, oncocytomalike AML, and lymphangiomyomatosis of the renal sinus.^3^ The most common subtype is classic AML, composed of a variable amount of adipose tissue, spindle, and epithelioid smooth muscle cells mixed within abnormal thick-walled blood vessels. On the other hand, epithelioid AML comprises sheets of pure epithelioid cells with immunoreactivity to melanogenesis markers and no adipocytes or abnormal blood vessels. Tumor cells can display notable nuclear atypia and necrosis. Epithelioid AML can be benign or malignant that has the potential for local recurrence and distant metastasis.^2,3^

PEComas can occur sporadically or with tuberous sclerosis complex (TSC). TSC is an autosomal dominant genetic disease that occurs due to the loss of tumor suppressor genes TSC1 or TSC2 that act as an inhibitor of the rapamycin (mTOR) signaling pathway. Therefore, they have a crucial role in regulating cell growth, proliferation, autophagy, and protein and lipid synthesis. The clinical manifestations include mental retardation, seizures, and the development of mostly benign tumors in multiple organ systems, including the brain, heart, kidneys, and lungs. Renal cell carcinoma occurs in 2–3% of the TSC population, including children, and can be multicentric. Alterations of the TSC genes have been confirmed in many PEComas, either sporadic or associated with TSC.^1,2,4,5^

The imaging features of PEComas are non-specific and have not been widely described in previous reports. On CT and MR scans, the tumors are of homogeneous texture, most often of well-demarcated outlines and of variable sizes. They show significant enhancement in contrast-enhanced CT or MRI in arterial and portovenous phases that become mildly hypodense/hypointense in delayed phases. No macroscopic fat could be appreciated in contrast to classic AML. Aggressive behavior in the form of invasion of renal vein and renal fascia has been described in malignant PEComas. On ultrasound, they have heterogeneous echotexture, and large tumors can show cystic/necrotic areas.^3,6,7^

Immunohistopathological evaluation of epithelioid AML is characterized by sheets or nests of large polygonal epithelioid cells with copious eosinophilic to occasionally clear cytoplasm, often with prominent nucleoli, and may include multinucleated and notably pleomorphic forms. PEComas usually show immunoreactivity for melanocytic (HMB-45 and/or melan-A) and smooth muscle (actin and/or desmin) markers, which help confirm the diagnosis. Generally, these tumors are benign, but some cases have malignant behavior and simulate high-grade sarcoma.^8^ The poor prognostic factors include tumor size of more than 5-centimeter, infiltrative growth pattern, high nuclear grade (more than 1 mitotic figure/50 HPF), atypical mitotic figures, and coagulative cell necrosis. Malignant PEComas should be suspected when there are two or more worrisome histologic features.^9^

Surgical resection and chemotherapy were the treatment options for PEComas. However, in recent years, there have been significant advances regarding TSC genes and their essential role in regulating the Rheb/mTOR/p70S6K pathway. Sirolimus and Everolimus, which are TOR inhibitors, seem to provide encouraging results and significant clinical outcomes in patients with PEComas. Furthermore, promising laboratory results are also noticed in the case of non-TSC and malignant PEComas.^4^

## Learning points

- PEComas are rare tumor especially in pediatric age group.
- PEComas should be included in the differential diagnosis of any soft tissue masses in patients with TSC.
- Lack of specific imaging characteristics is challenging for radiologist.
